# Middle Ear Primary Oncocytic Schneiderian Papilloma: A Case Report

**DOI:** 10.22038/IJORL.2022.61033.3142

**Published:** 2022-09

**Authors:** Mohamad Reza Afzalzadeh, Shirin Taraz Jamshidi, Zahra Zohani

**Affiliations:** 1 *Sinus and Surgical Endoscopic Research Center, Faculty of Medicine, Mashhad University of Medical Sciences, Mashhad, Iran.*; 2 *Cutaneous Leishmaniosis Research Center, Mashhad University of Medical Sciences, Mashhad, Iran.*; 3 *Department of Pathology, School of Medicine, Mashhad University of Medical Sciences, Mashhad, Iran.*

**Keywords:** Middle ear, Oncocytic, Papilloma, Schneiderian.

## Abstract

**Introduction:**

Chronic otitis media is a significant health problem, but middle ear and mastoid neoplasms, either benign or malignant, are extremely rare.

**Case Report:**

Here is a report from a 51-year-old female who presented persistent otorrhea with an aural polyp. The patient was operated on with the probable diagnosis of cholesteatoma. During surgery, a fragile mass was discovered, and histopathologic examination reported the diagnosis of a primary oncocytic Schneiderian papilloma. Microscopically it has pseudostratified epithelium of columnar cell epithelium with eosinophilic granular cytoplasm and hyperchromatic nuclei. The treatment of choice for Schneiderian papillomas is complete surgical removal.

**Conclusions:**

Although very rare, oncocytic Schneiderian papilloma should be considered a differential diagnosis of ear neoplasms such as auditory canal polyps.

## Introduction

Schneiderian papilloma is a benign neoplasm of the sinonasal respiratory mucosa. Most cases are seen in adults, but can also occur in children.

There are three distinct clinicopathologic variants of Schneiderian papilloma: exophytic, inverted, and oncocytic. 

Oncocytic papilloma is the least common and usually, occurs in the sinonasal area, although it is rarely reported in the middle ear (1).

Here we prepared a report from a primary, middle ear oncocytic Schneiderian papilloma as an educational example and a reminder to consider it a differential diagnosis of auditory canal polyps.

## Case Report

**Fig 1:**
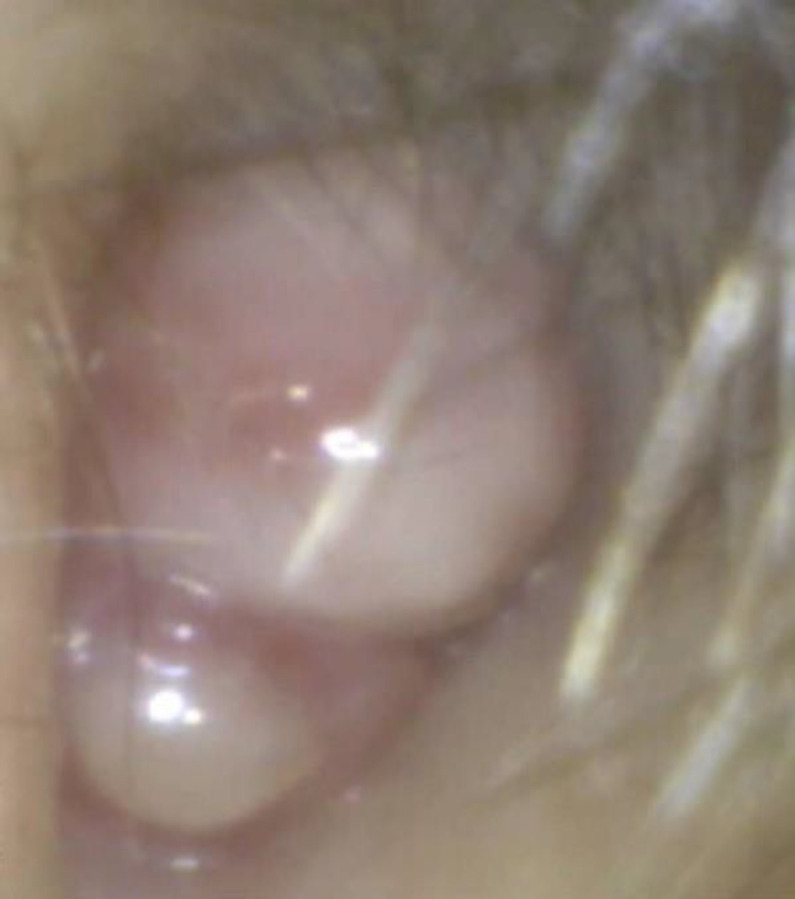
Otoscopy showing the polypoid tissue occupying the external auditory canal

A 51-year-old woman complained of a polypoid lesion in the external auditory canal. She suffered from purulent otorrhea nonresponsive to medical treatment and hearing difficulty in the last 12 months. Otomicroscopy showed a bulky polypoid tissue blocking the canal completely and therefore obscuring the tympanic membrane (Fig.1). 

**Fig 2:**
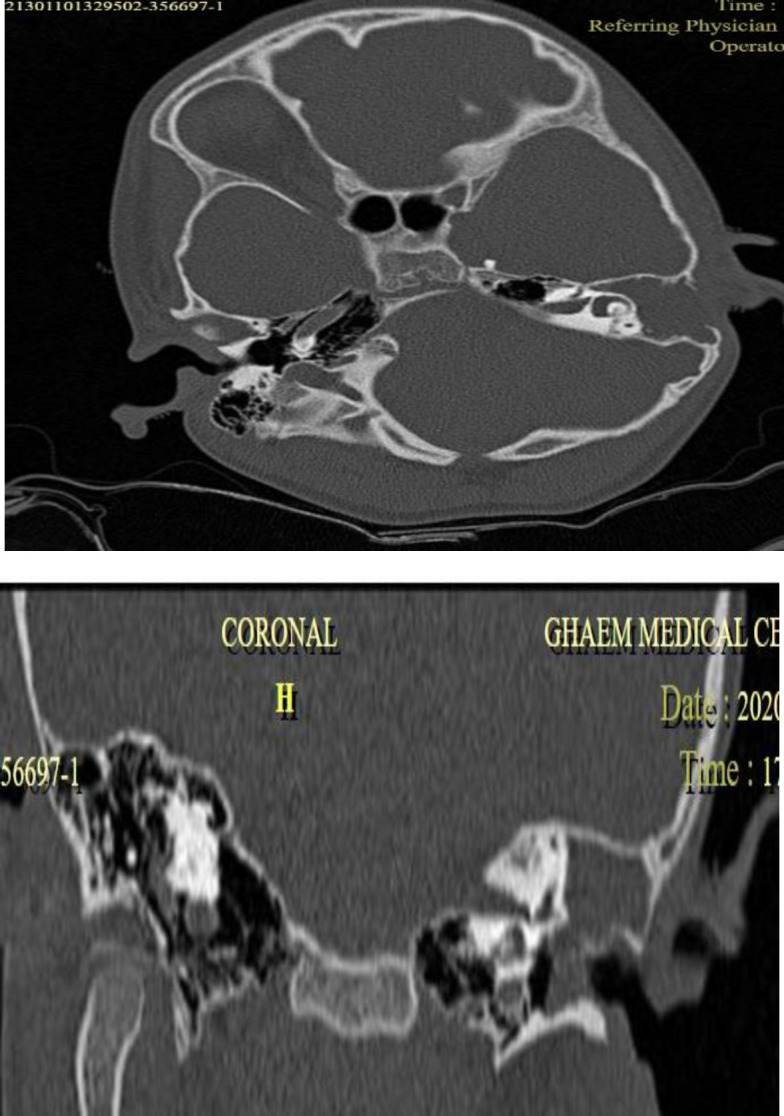
A- Axial temporal computed tomography scan illustrating soft tissue density in left middle ear that coalesces the mastoid cells and erodes the lateral cortex of mastoid B- Coronal temporal computed tomography scan illustrating soft tissue density in middle ear and middle external auditory canal

Computed tomography scan of temporal bone showed a soft tissue density in the external, middle ear, and mastoid with the destruction of ossicles and mastoid air cells and lateral cortex of mastoid (Fig.2). 

The patient was operated with the probable diagnosis of cholesteatoma. Mastoidectomy was performed, and, fragile hemorrhagic tissue was found in the mastoid antrum and middle ear with the destruction of ossicles. The canal wall down approach was chosen for eradication of infection. Histologic diagnosis was reported to be inverted oncocytic Schneiderian papilloma.

The postoperative course was good with unremarkable two-week, three-, and six-month follow-ups. The nasal endoscopy was normal with no remarkable lesion. The cavity was clear of pathology six months after surgery, and no recurrence was detected.

## Discussion

The external auditory canal polyp in a patient, with chronic otitis media is approached as cholesteatoma until proven otherwise. The patient in this case report had an aural polyp and a history of otorrhea for ten years with a CT scan diagnosis of cholesteatoma. We performed surgery without previous biopsy as routine, and surprisingly the histologic result was oncocytic Schneiderian papilloma of the middle ear.

Ear polyps are granular tissues usually found at the connection of cholesteatoma and bone. However, other ear lesions, including malignancies, may also appear as a mass of the external auditory canal. So, this is not surprising that oncocytic Schneiderian papilloma was not considered the initial diagnosis. Schneiderian papilloma is an unusual diagnosis, estimated at an annual rate of 2.3 per 100,000 people (2). A total of 58 cases of otologic involvement have been described in the literature (including our case). The oncocytic subtype is a rare neoplasm in the sinonasal area. The prevalence was 6% of all Schneiderin papillomas (3). 

Formerly, only one case of oncocytic Shneiderian papilloma of the middle ear was reported (4).

**Fig 3:**
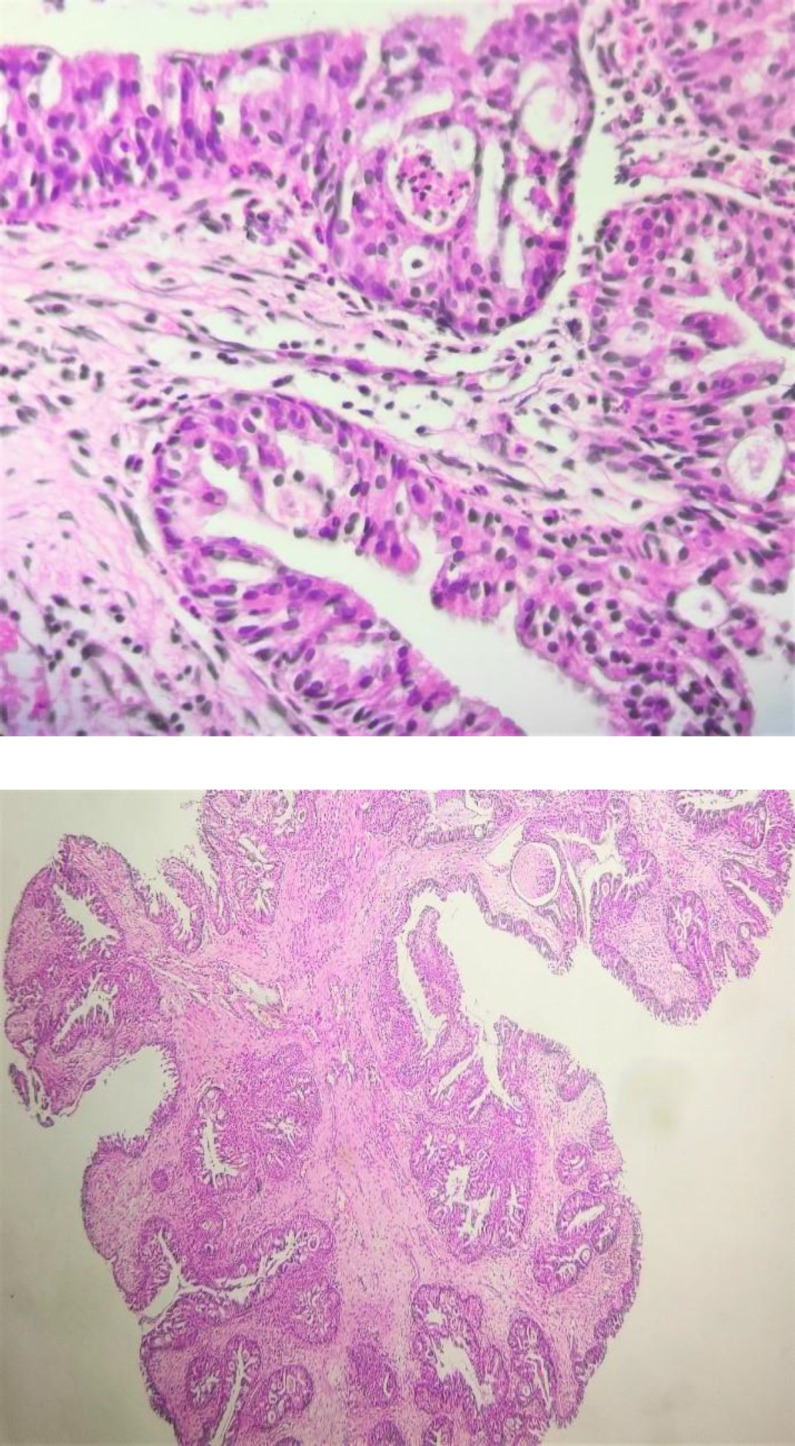
Microscopic view of oncocytic schneiderian papilloma. A-predominantly endophytic epithelial growth pattern. B-high magnification shows epithelial proliferation consisting of oncocytic cylindrical cells with abundant eosinophilic and granular cytoplasm, intraepithelial mucin filled cysts with neutrophilic microabscess

Direct spread through the Eustachian tube seems to be the main cause of this disease (5). Two other theories are abnormal embryonic migration of the ectopic Schneiderin membrane into the middle ear mucosa and stimulation of chronic otitis media resulting in the development of Schneiderian mucosa (6-9). These theories could also be suggested for oncocytic subtypes originating in the middle ear. Macroscopically, Oncocytic Schneiderian papillomas are papillary or polypoid lesions with a red to brown color. Microscopically, they have multilayered columnar epithelial cells with exophytic or endophytic growth patterns and are composed of eosinophilic and granular cytoplasm, vesicular to hyperchromatic nuclei, and often inconspicuous nucleoli (Fig.3). 

Epithelial cells may have cilia. This lesion may have intraepithelial mucin-filled cysts with neutrophilic microabscess. The stromal component may be myxoid to fibrous with infiltration of inflammatory cells and variable degrees of vascularity (10).

The treatment of all Schneiderian papilloma is complete surgical removal, including the adjacent non-involved mucosa. Oncocytic and inverted subtypes may undergo malignant transformation. The incidence of this phenomenon for oncocytic variants ranges from 4% to 17% (11). Schneiderian papillomas in the ear and temporal bone are at greater risk for malignant transformation (33%) than papillae exclusively in the sinus area (2-8%) (12).

## Conclusion

Although incidence in the middle ear is rare, oncocytic Schneiderian papilloma should be assumed as a differential diagnosis of middle ear neoplasms.
